# Association between a Single Donor *TARC/CCL17* Promotor Polymorphism and Obstructive Chronic Lung Allograft Dysfunction after Lung Transplantation

**DOI:** 10.3389/fimmu.2017.01109

**Published:** 2017-09-06

**Authors:** Kevin Budding, Jessica van Setten, Eduard A. van de Graaf, Oliver A. van Rossum, Tineke Kardol-Hoefnagel, Erik-Jan D. Oudijk, C. Erik Hack, Henderikus G. Otten

**Affiliations:** ^1^Laboratory of Translational Immunology, University Medical Center Utrecht, Utrecht, Netherlands; ^2^Department of Cardiology, University Medical Center Utrecht, Utrecht, Netherlands; ^3^Department of Respiratory Medicine, University Medical Center Utrecht, Utrecht, Netherlands; ^4^Center of Interstitial Lung Diseases, St. Antonius Hospital, Nieuwegein, Netherlands; ^5^Department of Rheumatology, University Medical Center Utrecht, Utrecht, Netherlands; ^6^Department of Dermatology, University Medical Center Utrecht, Utrecht, Netherlands

**Keywords:** lung transplantation, thymus and activation-regulated chemokine, chronic lung allograft dysfunction, bronchiolitis obliterans syndrome, chronic rejection

## Abstract

Lung transplantation (LTx) outcome is hampered by development of chronic rejection, often manifested as the bronchiolitis obliterans syndrome (BOS). Low serum levels of thymus and activation-regulated chemokine (TARC/CCL17), a chemoattractant, measured during the first month post-LTx are predictive for BOS development. Since *TARC/CCL17* promotor polymorphisms correlate with serum TARC/CCL17 levels, we investigated seven single-nucleotide polymorphisms (SNPs) within this region and their potential association with LTx outcome. We analyzed donor and patient SNP configurations and haplotypes and observed a trend between a donor SNP (rs223899) configuration and patient TARC/CCL17 serum levels post-LTx (*p* = 0.066). Interestingly, this SNP configuration in patients did not show any correlation with pre-LTx TARC/CCL17 serum levels (*p* = 0.776). Survival analysis showed that receiving a graft from a donor heterozygous for rs223899 has a disadvantageous impact on transplantation outcome. When stratified per donor SNP genotype, patients receiving a transplant from a heterozygous donor showed a lower BOS-free survival (*p* = 0.023) and survival rate (*p* = 0.0079). Since rs223899 is located within a NFκB binding site, heterozygosity at this position could result in a reduced TARC/CCL17 expression. Our data indicate that a single *TARC/CCL17* promotor SNP in the donor correlates with lower serum TARC/CCL17 levels measured 1 month after LTx and affects clinical outcome after LTx.

## Introduction

For patients suffering from end-stage lung disease, lung transplantation (LTx) can be the final treatment modality. Currently, 5-year survival after LTx is 50%, predominantly due to the development of chronic lung allograft dysfunction (CLAD) (1). CLAD can present an obstructive (bronchiolitis obliterans syndrome, BOS) and a restrictive form (restrictive allograft syndrome, RAS) (2). CLAD pathogenesis is poorly understood; however, various donor and patient risk factors associated with disease development have been identified, particularly regarding development of BOS (3, 4). A clinical diagnosis of BOS is often made using a 20% decline of the forced expiratory volume in 1 s compared to baseline in the absence of any other disease etiology (5). Thus, a clinical diagnosis is made at the time that obliterative bronchiolitis has fully developed. To prevent BOS, novel biomarkers reflecting preclinical development identifying patients at risk early after transplantation are urgently needed (6).

Thymus and activation-regulated chemokine (TARC/CCL17) is a chemoattractant, which is secreted by various cell types, including endothelial cells, dendritic cells, keratinocytes, bronchial epithelial cells, and fibroblasts (7–10). It mainly functions as a chemoattractant for Th2 cells *via* the interaction with its receptor CCR4 (11, 12). TARC/CCL17 serum levels are associated with various types of lung diseases including idiopathic pulmonary fibrosis (13) and eosinophilic pneumonia (14), and as risk marker for lung cancer (15). Interestingly, previous results from our group have shown that serum levels of TARC/CCL17 in the first month post-transplantation are predictive for BOS development after LTx (16).

The *TARC/CCL17* gene is located on chromosome 16q13, in near proximity of the CCR4 interacting chemokine *CCL22* and *CX3CL1* (17). TARC/CCL17 expression is controlled by multiple pro-inflammatory cytokines, including tumor necrosis factor-α, interferon (IFN)-γ, interleukin (IL)-1, and IL-4 (18). The transcriptional regulation of the TARC/CCL17 gene has partly been elucidated. Both the transcription factors STAT6 and NFκB have binding sites in the promotor region of *TARC/CCL17* (18, 19). Several single-nucleotide polymorphisms (SNPs) in the *TARC/CCL17* promotor region correlate with serum levels of TARC/CCL17 and are associated with a risk for Kawasaki disease and different allergic diseases (20–22).

As low early post-transplantation serum levels of TARC/CCL17 predict a risk for post-LTx BOS, we hypothesized that *TARC/CCL17* polymorphisms may be correlated to outcome after LTx. In this study, we genotyped and analyzed several SNPs in the TARC promotor region of patients undergoing LTx as well as in that of the donor. We show that a single donor SNP configuration in the promotor region of *TARC/CC17* of the donor correlates with recipient TARC/CCL17 serum levels and relates to BOS development and overall survival after LTx.

## Patients and Methods

### Patients

A total of 144 patients undergoing LTx between January 2004 and March 2013 in the Heart Lung Center of the University Medical Center Utrecht, The Netherlands, were included in this retrospective study. Written informed consent was obtained from all study participants, and the study was approved by the medical ethical committee of the University Medical Center Utrecht (METC 06-144), and all methods were carried out in accordance with the approved guidelines. Post-transplantation follow-up therapy was standardized and consisted of tacrolimus, prednisolone, and mofetil mycophenolate. In the first year after transplantation, spirometry was performed every week during the first 3 months reducing it to every 4 weeks after a year. Lung volumes were routinely assessed every 6 months and on indication when spirometry or X-ray changed. Patients at high risk for cytomegalovirus (CMV) or Epstein–Barr virus (EBV) activation, i.e., CMV- or EBV-negative patients transplanted with a EBV- or CMV-positive donor, were treated with valganciclovir for 6 months after transplantation. A clinical diagnosis of BOS was made when FEV_1_ had declined by 20% of more compared to baseline (5). Since surveillance biopsies were not performed, acute rejection (AR) was defined as a spontaneous decline of lung function that was reversed after steroid pulse treatment and for which other causes of lung function decline were excluded.

Prior to transplantation, blood was obtained from donor and patient, as well as a spleen samples from the donor. Mononuclear cells from patient and donor samples were isolated using Ficoll-paque (GE Healthcare, Little Chalfont, UK), which were then aliquoted and stored in liquid nitrogen until further use. In addition, serum from the patient was collected and stored at −80°C.

### DNA Extraction

Frozen mononuclear cells were used for DNA isolation *via* the MagnaPure Compact System (Roche Diagnostics, Switzerland) according to protocol. Cell samples were thawed at 37°C, dissolved in 9 ml RPMI-1640 (Lonza, Basel, Switzerland) supplemented with 20%, v/v, fetal bovine serum (Bodinco, Alkmaar, The Netherlands), and centrifuged for 10 min at 1,800 RPM. Prior to DNA extraction, cells ware dissolved in phosphate-buffered saline (Sigma-Aldrich, USA) at a concentration of 5 × 10^6^ cells/ml. After DNA extraction, both concentration and purity were analyzed using the NanoDrop™ system (Thermo Fischer Scientific, Waltham, MA, USA).

### SNP Selection and Genotyping

Six SNPs (rs223895, rs223897, rs223898, rs223899, rs223900, and rs229827) in the promotor region of TARC/CCL17 that are frequent in the western European population were selected from the HapMap (http://hapmap.ncbi.nlm.nih.gov/) and the Ensemble databases (23). We also analyzed the configuration of rs229828, which configuration has previously been associated with CCCL17/TARC serum levels (20). Samples were genotyped using the Affymetrix “TxArray” (24, 25) containing 767,203 variants, and stringent quality control (QC) was conducted to remove low-quality SNPs and samples. Samples with a missing rate >3% were removed. We created a subset of high-quality, independent SNPs with missing rate <1%, Hardy–Weinberg *p* > 0.001, minor allele frequency >0.1, and LD pruning leaving no SNP pairs with *r*^2^ > 0.2. Using this subset, we removed samples with heterozygosity >2 SD from the mean of all samples, related samples (keeping only one samples of each pair with proportion of IBD > 0.2), and samples of non-European ancestry [based on principle component analysis using the 1000 Genomes Project (Phase 1) populations as reference (26)]. SNPs were removed if they had a missing rate >5%, Hardy–Weinberg *p* < 0.01, or if they were monomorphic. After QC, 543,637 SNPs and 133 patients and 131 donor samples remained. Untyped SNPs were imputed using a combined reference panel of the 1000 Genomes Project (Phase 3) (27) and the Genomes of the Netherlands (v5) (28). Samples were first phased with SHAPEIT (29) and then imputed with IMPUTE v2 (30).

### Measurement of Serum TARC/CCL17 Concentrations

Serum concentrations of TARC/CCL17 were determined *via* a solid-phase ELISA kit (R&D systems, Minneapolis, MN, USA) according to protocol. Briefly, wells were first incubated with serum samples for 2 h, then with conjugate for 1 h, and finally with substrate. From OD450 values, levels were calculated by reference to a standard curve. Serum samples were briefly centrifuged prior to analysis. All samples where measured in duplicate. Inter- and intra-assay variability’s of the assay were 8.3 and 4.4%, respectively.

### Statistics

All statistical analyses were performed using GraphPad Prism version 6.02 (GraphPad Software Inc., San Diego, CA, USA) and SPSS version 21 (IBM Corp., Armonk, NY, USA). Data were tested for Gaussian distribution *via* the D’Agostino and Pearson omnibus normality test. Normally distributed data are represented as mean value ± SEM whereas data not following a Gaussian distribution are represented as median ± interquartile range. Depending on the distribution of the data, differences between groups were analyzed with the unpaired *t* test or the Mann–Whitney test, indicated in the respective figure legend. Differences in categorical data were analyzed using the Fischer’s exact test and in continuous variables *via* ANOVA. Survival analyses were conducted using Kaplan–Meier analysis with both BOS incidence and overall survival as endpoint parameters. A Cox-regression model was used for multivariate analysis including known risk factors in patients and donors for BOS development. A *p*-value <0.05 was considered to be statistical significant.

## Results

### Patient Demographics

From the total cohort of 144 patients transplanted in our center, 65 were treated with LTx because of chronic obstructive pulmonary disease, 42 because of cystic fibrosis, 36 because of interstitial lung disease, and 1 patient was diagnosed with pulmonary vascular disease prior to transplantation. Besides the fact that BOS + patients were slightly older at the time of transplantation, no significant demographic and clinical differences were observed between BOS+ and BOS− groups (Table 1). During transplantation follow-up, 44 patients developed BOS. No RAS was observed. In total, 44 patients deceased during the study period, whereas 20 patients presented with one or more AR episodes.

**Table 1:** Clinical and demographic parameters of lung transplant patients and donors.

	All	Non-BOS	BOS	*p*-Value
**Patients**				

**Total number**	144	100	44	

**Gender**				
Male	69	52	21	0.560
Female	75	48	23	

**Mean age (years)**	46 ± 13	44 ± 14	50 ± 11	*0.026*
**Mean follow-up (months)**	61.2 ± 36.8	59.2 ± 39.4	65.6 ± 30.2	0.341

**Primary disease**				
CF	65	40	25	0.247
COPD	42	33	9	
ILD	36	26	10	
PVD	1	1	0	

**Infection**				
EBV high risk	14	7	7	0.115
CMV high risk	32	21	11	0.456

**Type of graft**				
Bilateral	112	81	31	0.119
Single	32	19	13	

**Episode of acute rejection**	20	14	6	0.495

**Ischemic times (min)**				
Bilateral	312.3 ± 188.9	321.4 ± 216.9	288.6 ± 73.8	0.426
Single	244.1 ± 53.5	238.2 ± 48.8	238.7 ± 73.0	0.314

**Donors**				

**Gender**				
Male	65	46	19	0.449
Female	79	54	25	

**Donor age (years)**				
Mean age	45 ± 14	44 ± 15	47 ± 14	0.184
>60	17	12	5	0.579

**Donor type**				
HB	116	20	8	0.497
Non-HB	28	80	36	

**Smoking**				
Yes	52	35	17	0.407
No	92	65	27	

*Cohort overview of both patients and donors subdivided for the incidence of BOS after LTx*.

*BOS, bronchiolitis obliterans syndrome; CF, cystic fibrosis; COPD, chronic obstructive pulmonary disease; ILD, interstitial lung disease; PVD, pulmonary vascular disease; EBV, Epstein–Barr virus; CMV, cytomegalovirus; HB, heart beating; LTx, lung transplantation*.

### *TARC/CCL17* Promotor Polymorphisms

All extracted DNA samples from patient/donor couples were analyzed on the Affymetrix-based TxArray and selected SNPs were imputed as described in Section “Patients and Methods.” After stringent pre- and post-imputation QC, including deviation from Hardy–Weinberg equilibrium, sample and SNP missingness, heterozygosity checks, and principle component analyses (data not shown) (31), 133 patients and 131 donor could be genotyped for the selected *TARC/CCL17* promotor SNPs (92.4 and 91.7% of the total cohort, respectively). From all samples identified, SNP genotypes were stratified per haplotype. Table 2 describes these results as well as haplotype and genotype frequencies of the individual SNPs. We observed no significant differences in either SNP or haplotype distribution between patients and donors. Also, the genotype frequencies of the selected *TARC/CCL17* SNPs were in concordance with frequencies found in the HapMap and the Ensemble databases.

**Table 2:** Genetic configuration of selected *TARC/CCL17* promotor polymorphisms.

Haplotypes							Patients		Donors	
rs223895	rs223897	rs223898	rs223899	rs223900	rs223827	rs223828				
		
Y	Y	K	K	Y	Y	Y	*n*	%	*n*	%
C	C	T	G	C	T	C	42	0.316	41	0.318
Y	Y	K	K	Y	Y	C	26	0.195	32	0.242
Y	C	T	G	C	Y	C	17	0.128	15	0.114
C	C	T	G	C	Y	C	12	0.090	13	0.098
Y	Y	K	K	Y	Y	Y	8	0.060	7	0.053
T	Y	K	K	Y	C	C	7	0.053	9	0.068
Y	Y	K	K	Y	C	C	4	0.030	3	0.023
T	T	G	T	T	C	Y	3	0.023	4	0.030
Y	Y	K	G	Y	Y	C	3	0.023	2	0.015
T	T	G	T	T	C	C	2	0.015	1	0.008
T	T	G	K	T	C	C	2	0.015	n.o	n.o
T	T	G	T	T	C	T	2	0.015	n.o	n.o
T	Y	K	G	Y	C	C	1	0.008	n.o	n.o
T	Y	K	K	Y	C	Y	1	0.008	n.o	n.o
T	C	T	G	C	C	C	1	0.008	n.o	n.o
Y	Y	K	G	Y	C	C	1	0.008	n.o	n.o
Y	Y	K	K	Y	C	Y	1	0.008	2	0.015
C	C	T	G	C	C	C	n.o	n.o	1	0.008
T	Y	K	K	Y	Y	C	n.o	n.o	1	0.008
C: 0.413	C: 0.538	G: 0.053	G: 0.564	C: 0.538	C: 0.170	C: 0.892	133		131	
T: 0.129	T: 0.053	T: 0.538	T: 0.045	T: 0.053	T: 0.314	T: 0.008				
Y: 0.458	Y: 0.406	K: 0.409	K: 0.391	Y: 0.409	Y: 0.515	Y: 0.10				

*Overview of incidence and frequency of single-nucleotide polymorphism (SNP) configurations (vertically) and observed haplotypes (horizontally) for both patients and donors. SNPs are displayed according to their relative distance from the transcription initiation site. All observed SNP frequencies were in concordance with the Ensemble database*.

### rs223899 Influences Serum TARC/CCL17 Concentrations Post-Transplantation

**Figure 1:**
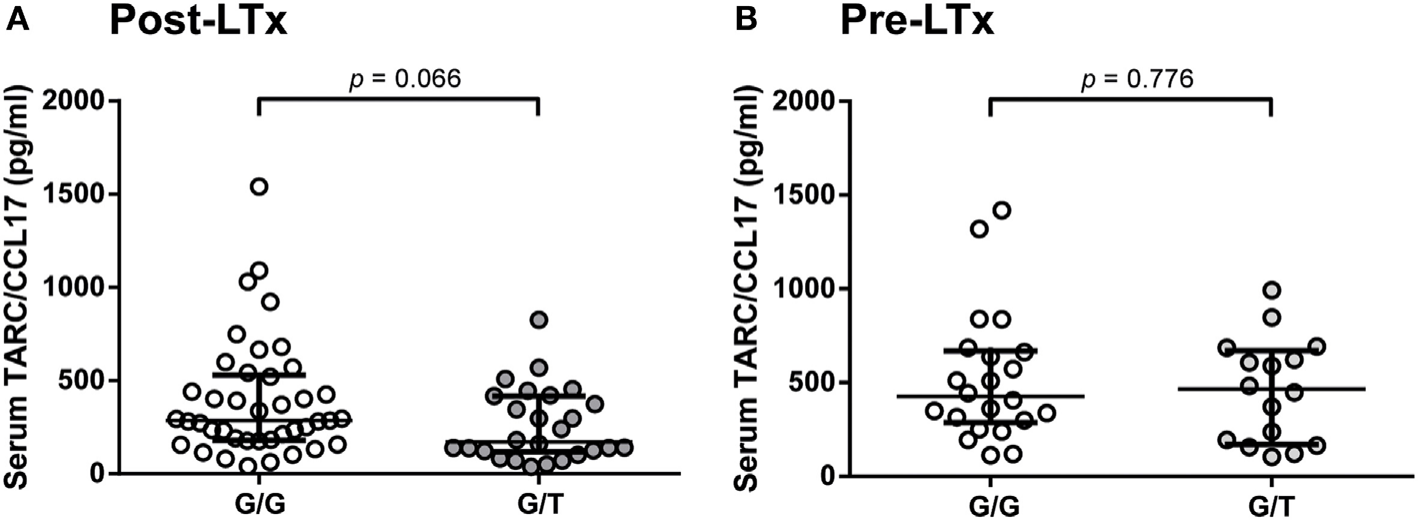
Donor rs223899 correlates with serum thymus and activation-regulated chemokine (TARC/CCL17) concentrations post-lung transplantation (LTx) LTx but not pre-LTx. **(A)** Serum TARC/CCL17 levels were measured in LTx patients 1 month after LTx stratified for the configuration of donor single-nucleotide polymorphism (SNP) rs223899 (homozygous, G/G, 286.0 pg/ml vs. heterozygous, G/T 170.5 pg/ml). A trend toward significance (*p* = 0.066) was observed with increased TARC/CCL17 serum concentrations in patients who received a graft genotyped homozygous for SNP position rs223899. Non-Gaussian distribution, median ± interquartile range, Mann–Whitney test, homozygous *n* = 41, heterozygous *n* = 26. **(B)** Serum TARC/CCL17 levels were also assessed pre-LTx in patients based on serum availability. No differences in serum TARC/CCL17 could be observed when patients were stratified for the respective rs223899 SNP genotype. Non-Gaussian distribution, median ± interquartile range, Mann–Whitney test, homozygous *n* = 22, heterozygous *n* = 16.

From a subset of 67 representative patients (no differences on clinical and demographic parameters compared to the total cohort), serum samples obtained during the first month after transplantation were analyzed for TARC/CCL17 levels, based on serum sample availability. The relation between both donor and patient haplotypes and SNP genotypes, and serum TARC/CCL17 levels were then analyzed. The strongest association was observed between donor SNP rs223899 and serum levels. Serum TARC/CCL17 levels in patients with lungs from donors with the homozygous (G/G) SNP configuration of rs223899, tended to be higher than those in patients with a lung from a donor with heterozygous (G/T) configuration (*p* = 0.066, Figure 1A). Notably, serum TARC/CCL17 levels before transplantation were not different in these patient groups (*n* = 38, *p* = 0.776, Figure 1B).

### Donor SNP rs223899 Influences the Clinical Outcome after LTx

Since we observed that patient serum TARC/CCL17 levels correlate with the configuration of donor SNP rs223899, and decreased serum levels of TARC/CCL17 predict a higher risk for BOS development after transplantation (16), we analyzed the genotyped donor haplotypes and individual donor SNP configurations in a Kaplan–Meier survival analysis. For overall survival, all 131 patients for which the imputed donor SNP passed QC were included. For the analyses of BOS development, we excluded patients who had deceased within the first 4 months after transplantation or from whom SNP analysis did not pass QC, resulting in the inclusion of 122 patients.

**Figure 2:**
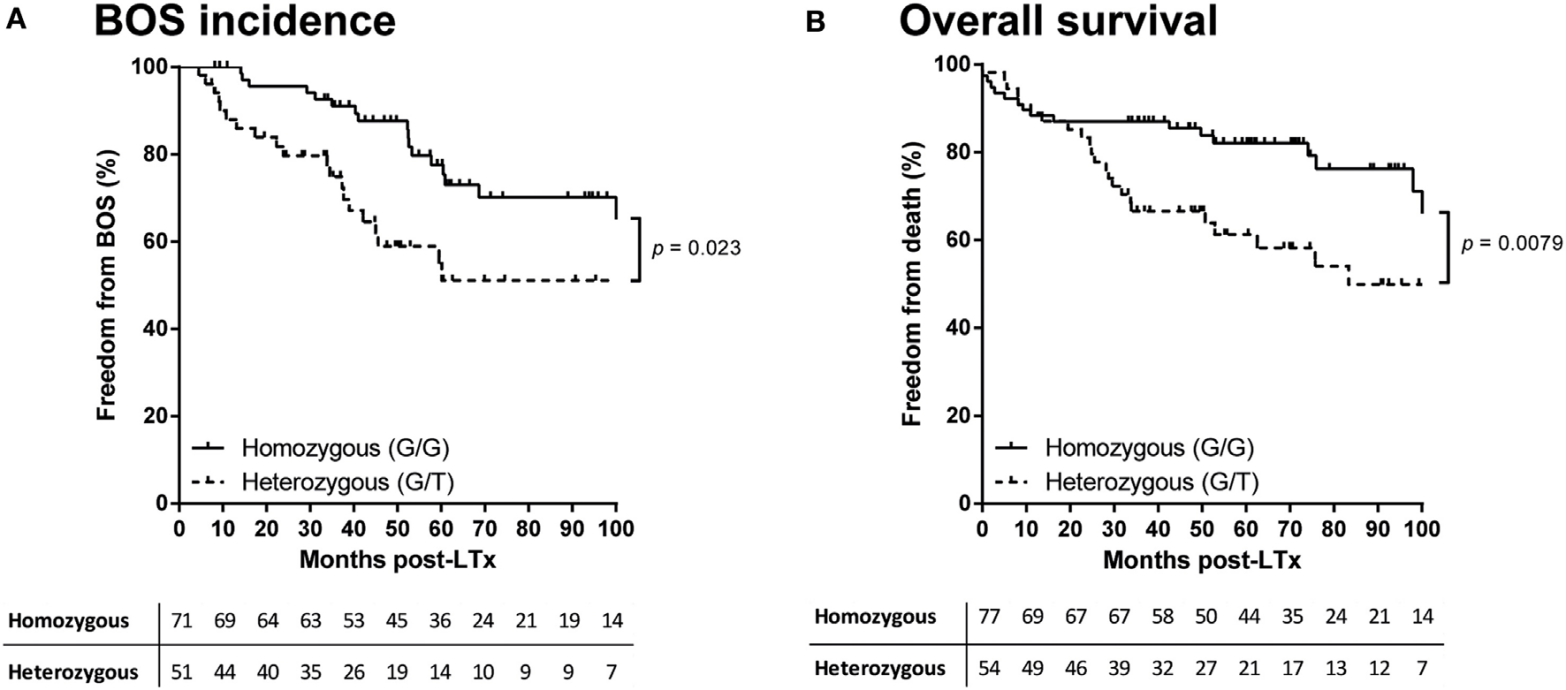
Donor rs223899 affects clinical outcome after lung transplantation (LTx). **(A)** Kaplan–Meier analysis on bronchiolitis obliterans syndrome (BOS) incidence after LTx. Patients were stratified according to the single-nucleotide polymorphism (SNP) configuration of rs223899 in the received allograft. Patients who received a graft genotyped as heterozygous (G/T) for this specific SNP have a lower BOS-free survival rate measured over the first 100 months after transplantation (*p* = 0.023). Lower table represents numbers at risk. All 131 patients for which the imputed donor SNP passed quality control (QC) were included. **(B)** Kaplan–Meier analysis on survival after LTx. Patients were stratified as mentioned earlier. Additional to an increase of chronic rejection after LTx, stratification of LTx patients for receiving a grafted organ genotyped heterozygous (G/T) at SNP position rs223899 resulted in a lower survival rate post-LTx (*p* = 0.0079). Lower table represents numbers at risk. Patients who had deceased within the first 4 months after transplantation or from whom SNP analysis did not pass QC were excluded, resulting in the inclusion of 122 patients. Log-rank test used in both analyses.

In total, six different donor haplotypes had a frequency above 5% and were analyzed for correlation to outcome after LTx. None of the donor or patient haplotypes of the seven selected *TARC/CCL17* SNPs showed a correlation with either AR episodes, BOS incidence, or survival after LTx (data not shown). In contrast, we observed a significant difference in the development of chronic rejection when patients were stratified by donor SNP rs223899 genotype. Of the patients who received a transplant from a heterozygous (G/T) donor at position rs223899, 50% remained free from BOS within the first 100 months after transplantation. This percentage was significantly higher, 75%, in the patients who had received a graft from a homozygous (G/G) donor (*p* = 0.023, Figure 2A). This was confirmed in a multivariate analysis using a Cox proportional-hazards model for BOS development, which included both donor and patient risk factors for BOS development, and designated the risk variant of rs223899 as a significant predictor for the development of BOS after LTx (*p* = 0.018, Table 3). Also, this nucleotide substitution in the promotor region of *TARC/CCL17* in the donor correlated with a lower survival rate of recipients’ post-transplantation (50 vs. 80%, respectively, *p* = 0.0079, Figure 2B). None of the other individual donor SNPs correlated with BOS development or survival after LTx.

**Table 3:** Multivariate analysis on bronchiolitis obliterans syndrome (BOS) incidence in patients treated with lung transplantation.

	Hazard ratio (95% CI)	*p-*Value
Thymus and activation-regulated chemokine single-nucleotide polymorphism configuration	2.4 (1.2–5.0)	0.018
Donor age (≥60)	0.7 (0.2–2.5)	0.630
Donor smoking state	1.3 (0.6–2.6)	0.493
Cytomegalovirus reactivation	1.4 (0.6–3.3)	0.397
Epstein–Barr virus reactivation	2.2 (0.8–6.1)	0.131
Recipient age (≥60)	2.5 (1.1–5.7)	0.025
Episode of acute rejection	1.0 (0.4–2.5)	0.969

*Cox proportional-hazards model including both known patient and donor factors associated with BOS development*.

## Discussion

In this study, we analyzed the relation of both donor and patient *TARC/CCL17* genotypes to clinical parameters, but only found one single donor SNP to be correlated with BOS development. Interestingly, this specific promotor SNP configuration, heterozygosity for rs223899, also correlated with lower serum TARC/CCL17 levels, which is in concordance with previous observations that low serum TARC/CCL17 levels in the first month after transplantation are predictive for BOS development (16). We have also assessed patient–donor combinations stratified by rs223899 genotype. We observed higher concentrations of serum TARC/CCL17 in the first month after LTx when patients heterozygous for SNP rs223899 were transplanted with a homozygous donor. This was not observed in the other three combinations (patient/donor homozygous, patient/donor heterozygous, and patient homozygous/donor heterozygous).

We only analyzed SNPs that are frequent in the European population. Therefore, validation in an external cohort, and re-analysis of our findings in populations with other genetic backgrounds is expedient. In this cohort, BOS was diagnosed according to international guidelines. However, surveillance bronchoscopy and transbronchial lung biopsies are not performed, which is a limitation of this cohort. Unresolved infections or undiagnosed episodes of AR could be a possibility of persistent lung function decline. Also, due to cohort constraints, we were not able to discriminate between early and late BOS onset and potential *TARC/CCL17* SNP configurations.

Since TARC/CCL17 serum levels measured at month 1 post-LTx are increased and a predictor for BOS development (16), we investigated these serum levels in relation to the donor SNP configuration of rs223899. Unfortunately, bronchoalveolar lavage samples were not available. *TARC/CCL17* expression is controlled by various pro-inflammatory cytokines. The insignificant trend of lower serum TARC/CCL17 levels observed could be because of the immunosuppressive treatment which could potentially influence cytokine production and regulation of TARC/CCL17 production. Also, infections with community-acquired respiratory viruses could impact expression. However, we observed no differences in treatment regimen or the incidence of community-acquired respiratory viral infections in patients stratified for donor SNP genotype (Table S1 in Supplementary Material).

Most studies on the role of genetics in lung transplant complications have focused on the obstructive form of CLAD, BOS (32). These results are mainly obtained using patient DNA, illustrated by studies of Awad et al. concerning SNPs in IFN-γ and TGF-β1, in which the authors correlate gene polymorphisms with increased allograft fibrosis (33, 34). Also, an association between an IL-6 polymorphism and BOS development was observed (35). However, these findings could not be validated in independent cohorts (36). Recently, our group has shown that a SNP in the promotor region of complement regulatory protein CD59 in the donor correlates with a higher risk for chronic rejection after LTx (37). Furthermore, a specific donor MBL promotor haplotype has been associated with graft survival and BOS development after transplantation (38). Taken together, these data stress the potential importance of both patient and donor SNPs on the clinical outcome after LTx.

The correlation between rs223828, another *TARC/CCL17* promotor polymorphism, and protein serum levels has been described previously in a cohort of Japanese patients (10). This polymorphism was also found to be associated with atopy and asthma in children, as well as with higher circulating levels of TARC/CCL17 (21). We could not confirm the correlation of this SNP with serum TARC/CCL17 levels in our cohort of western European LTx patients, presumably due to low minor allele frequency in our patient cohort. Observations in patients suffering from Kawasaki disease have shown that rs223899 is associated with disease progression. However, individual SNPs, including rs223899, did not correlate with serum levels when stratified by genotype (22).

The genetic regulation of the *TARC/CCL17* gene has partly been elucidated. Two STAT6 binding sites have been identified at position -213/-223 and -177/-187 relatively to ATG upstream of Exon 1. Furthermore, a binding-motif for NFκB is present upstream of the two STAT6 binding sites (18). Interestingly, using a RSV-inducible mice epithelial cell model, Monick et al. have shown that optimal TARC expression is achieved *via* the combined activation of both transcription factors, which would involve the recruitment of CREB-binding protein/p300 *via* NFκB and is essential for STAT-mediated transcription (19). The identified SNP rs223899 lies within consensus binding sequence for NFκB (39). Thus, the heterozygous configuration of rs223899 could result in a less optimal NFκB binding, which would lead to a reduced expression of TARC/CCL17.

The role of serum levels of TARC/CCL17 in LTx outcome remains speculative. Bronchial epithelial cells have the potency to secrete large amounts of TARC/CCL17 when activated (7) and considering the small size of TARC/CCL17 (10.5 kDa), it seems logical to assume leakage from the allograft into the circulation that can subsequently be quantified in serum. Immunoregulatory functions have been attributed to TARC/CCL17 due to the presence of its receptor, CCR4, on a specific subset of regulatory T cells (40). A reduced secretion of TARC/CCL17 could lead to a diminished influx of regulatory T cells, which would result in less regulation of the overall immune response associated with transplant rejection (41, 42). Additional experiments are expedient to support this hypothesis.

In summary, our data indicate that heterozygosity for a single SNP in the promotor region of *TARC/CCL17* located within the consensus sequence of the binding site of transcription factor NFκB correlates with serum levels of the TARC/CCL17 protein. Low serum TARC/CCL17 levels are predictive for BOS development following LTx. In line with these observations, we show that patients who receive a heterozygous allograft for SNP rs223899 present with a higher BOS incidence and impaired survival after LTx.

## Ethics Statement

Informed consent was obtained from all study participants, and the study was approved by the medical ethical committee of the University Medical Center Utrecht (METC 06-144), and all methods were carried out in accordance with the approved guidelines.

## Author Contributions

KB, JS, TK-H, and OR performed the research; KB, JS, EG, OR, CH, and HO participated in data analysis; EG and E-JO contributed patient material; EG and HO participated in research design; KB, JS, EG, CH, and HO wrote the paper. All the authors provided final approval of the version to be published.

## Conflict of Interest Statement

The authors declare that the research was conducted in the absence of any commercial or financial relationships that could be construed as a potential conflict of interest.

## Abbreviations

AR, acute rejection; BOS, bronchiolitis obliterans syndrome; CF, cystic fibrosis; CLAD, chronic lung allograft dysfunction; COPD, chronic obstructive pulmonary disease; ILD, interstitial lung disease; LTx, lung transplantation; PVD, pulmonary vascular disease; RAS, restrictive allograft syndrome; SNP, single-nucleotide polymorphism; TARC, thymus and activation-regulated chemokine.
