# Supplementary material for: Association between a Single Donor TARC/CCL17 Promotor Polymorphism and Obstructive Chronic Lung Allograft Dysfunction after Lung Transplantation
Source: Front Immunol. 2017 Sep 6;8:1109. doi: 10.3389/fimmu.2017.01109 (PMC5592199; doi:10.3389/fimmu.2017.01109)
Supplement: Supplementary file 1 [file table_1.pdf]

Supplementary Table 1 Frequency distribution of various community acquired respiratory virusses and the incidence of Aspergillus infection in lung transplanta recipients stratified per donor TARC/CCL17 genotype (G, T, or G/T)

Pathogen	*p* -value
Aspergillus	0.645
Coronavirus	0.812
Rhinovirus	0.666
Human respiratory syncytial virus	0.168
Hepatitis B	0.588
Epstein-Barr virus	0.509
Herpes simplex virus 1	0.748
Influenza virus	0.111
Cytomegalovirus	0.400
Norovirus	0.733
Parainfluenza virus	0.574
Varizella zoster virus	0.948
Human metapneumovirus	0.622
Herpes simplex virus 2	0.305
Adenovirus	0.869
Hepatitis E	0.588
Rotavirus	0.588

Pearson's chi-squared test, SPSS version 21 (IBM)

tion
